# Supplementary material for: Design of a pH-Responsive Conductive Nanocomposite Based on MWCNTs Stabilized in Water by Amphiphilic Block Copolymers
Source: Nanomaterials (Basel). 2019 Oct 3;9(10):1410. doi: 10.3390/nano9101410 (PMC6835295; doi:10.3390/nano9101410)
Supplement: Supplementary file 1 [file nanomaterials-09-01410-s001.pdf]

Design of a pH-Responsive Conductive Nanocomposite Based on MWCNTs Stabilized in Water by Amphiphilic Block Copolymers

Frank den Hoed [1], Andrea Pucci [2], Francesco Picchioni [1] and Patrizio Raffa [1,*]

[1] Department of Chemical Engineering—Product Technology, University of Groningen, Nijenborgh 4, 9747 AG Groningen, The Netherlands; f.m.den.hoed@rug.nl (F.d.H.); f.picchioni@rug.nl (F.P.)
[2] Department of Chemistry and Industrial Chemistry, University of Pisa, Via Giuseppe Moruzzi 13, 56124 Pisa (PI), Italy; andrea.pucci@unipi.it
* Correspondence: p.raffa@rug.nl; Tel.: +315-0363-4465

Supporting information

Polymers synthesis and characterization (Section 3.1)

In the first step of the synthesis, the polystyrene macroinitiator was prepared in bulk at 100 ^{0}C. Methyl 2-bromopropionate (MBP) was used as initiator (I), Cu(I)Br as catalyst (C), PMDETA as ligand (L) and styrene as monomer (M). An analogous procedure was used for tert-butylacrylate. The results can be found in Table S1.

Table S1. Synthesis of polystyrene macroninitiator and polytert-butyl acrylate

Sample	I/C/L/M	Time (h)	M_n (GPC)	PDI	Styrene units (GPC)	tBA units (GPC)	Styrene units (NMR)
PS-Br	1/0.5/1/30	5	2880	1.08	26	-	24
PtBA	1/1.4/2.4/700	8	58200*	1.35	-	454	-

*dn/dc value assumed for tBA 0.0479[1]

The bromine terminated polystyrene was used as a macroinitiator in the second step of the reaction. The reaction mixture was diluted with 20 mL anisole and performed at 90 °C using Cu(I)Cl as catalyst. The results can be found in Table S2.

Table S2. Chain extension of PS-Br with tBA

Sample	I/C/L/M	Time (h)	Styrene units	tBA units (NMR)
PS-tBA (1)	1/2.2/4.1/200	5	26	81
PS-tBA (2)	1/2.2/2.7/500	5	26	226
PS-tBA (3)	1/2.2/2.7/1000	12	26	580

The obtained polymers were characterized by ^{1}H-NMR and GPC. For ^{1}H-NMR chloroform-d was used as a solvent. THF was used as eluent for GPC and toluene as a reference. To determine the molecular weight of the polystyrene by ^{1}H-NMR the area of styrene protons was compared to the initiator proton area (Figure S1). From this, the molecular weight could be calculated. The result only slightly deviated from the GPC results. The PtBA chain length was determined solely by GPC.

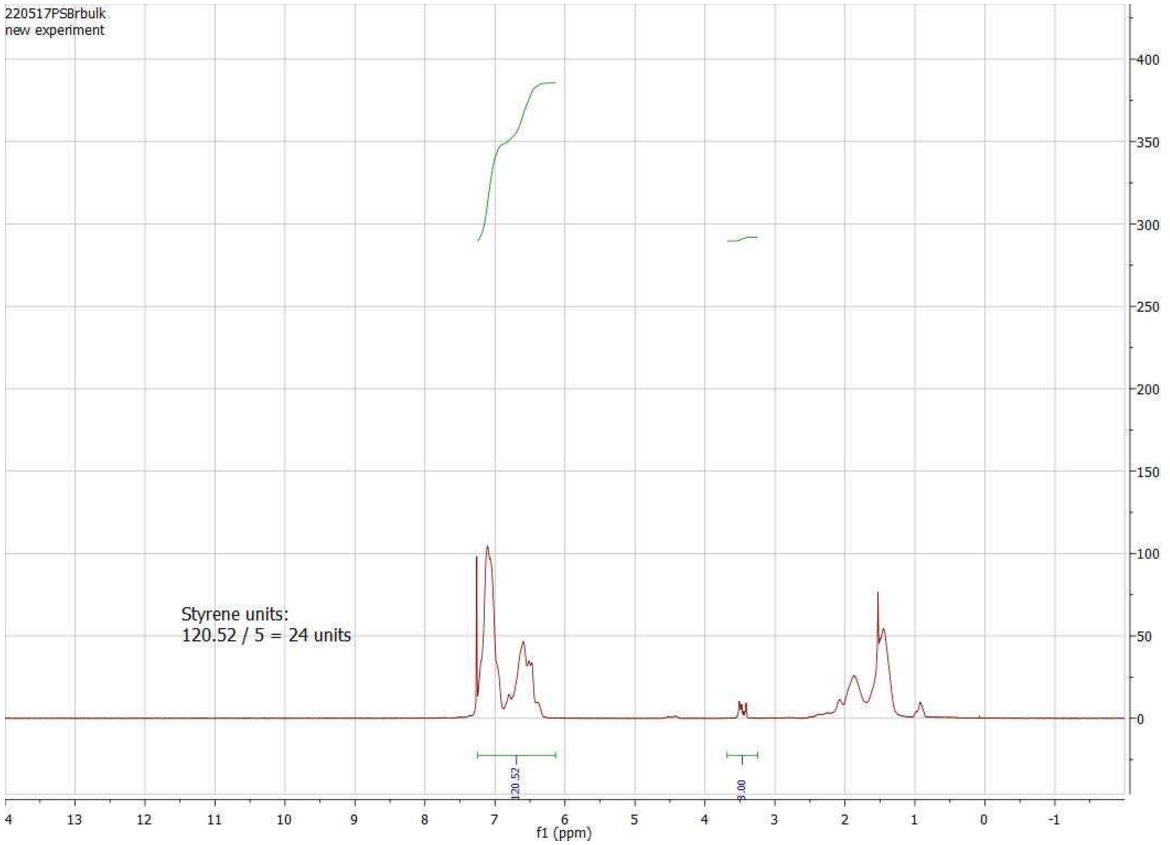

Figure S1. ^{1}H-NMR of PS-Br in $CDCl_3$

The copolymer lengths were determined solely by ^{1}H-NMR. Here, the area of the styrene protons was compared to the tert-butyl proton area. The backbone protons had to be deducted to calculate the chain extension length. One of the ^{1}H-NMR results is given in Figure S2, red graph.

The tert-butyl group was removed by hydrolysis yielding polyacrylic acid chains. The reaction was performed in 1,4-dioxane and an excess of HCl. The mixture was refluxed at 90 °C for 3 h. The resulting polymer was characterized by ^{1}H-NMR with d_6-DMSO as a solvent (Figure S2, green graph). The disappearance of the tBA peak (δ = 1.3 ppm) and the new peak at δ = 12.2 ppm show the success of the hydrolysis.

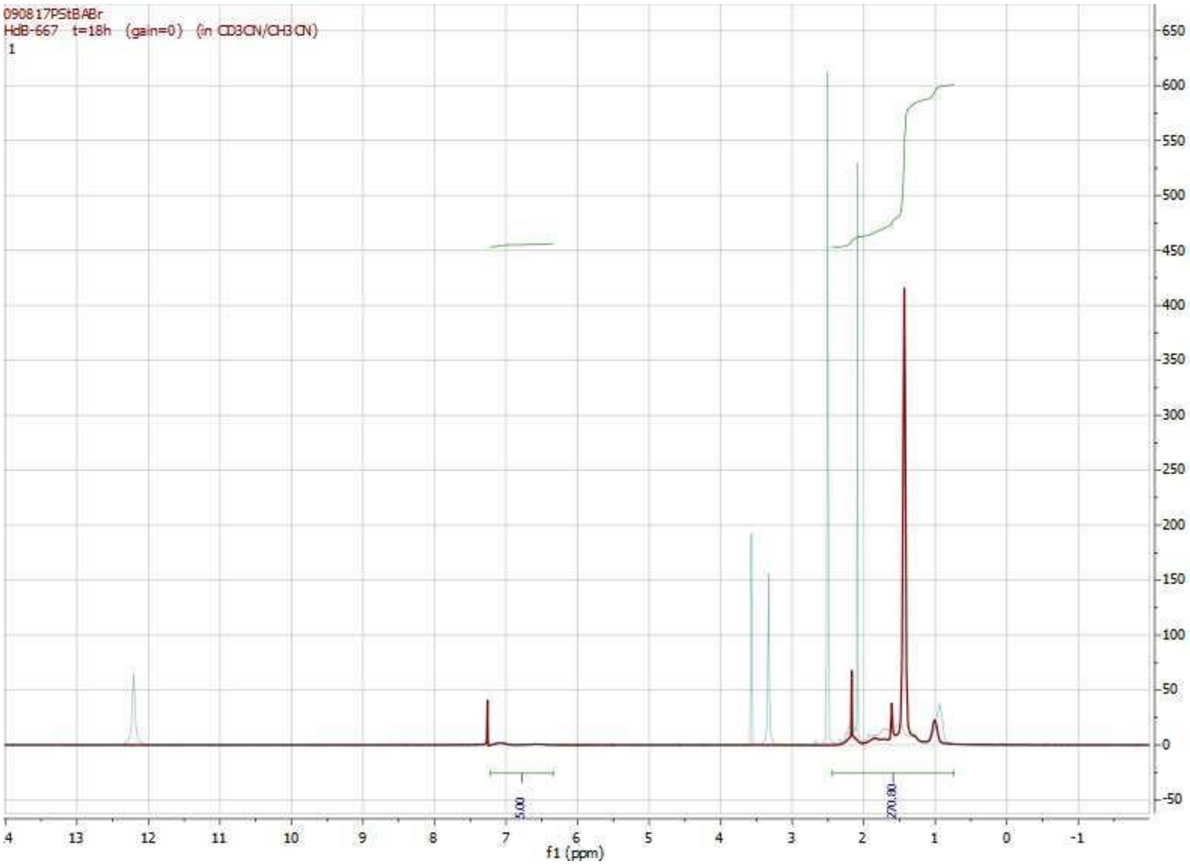

Figure S2. ^{1}H-NMR of PS-tBA (3) in $CDCl_3$ (red graph) and of PS26PAA580 in DMSO (green graph)

Polymer/MWCNT dispersion characterization via UV-vis spectroscopy (Section 3.2)

The content of MWCNT dispersed in solution was determined by UV-vis spectroscopy.

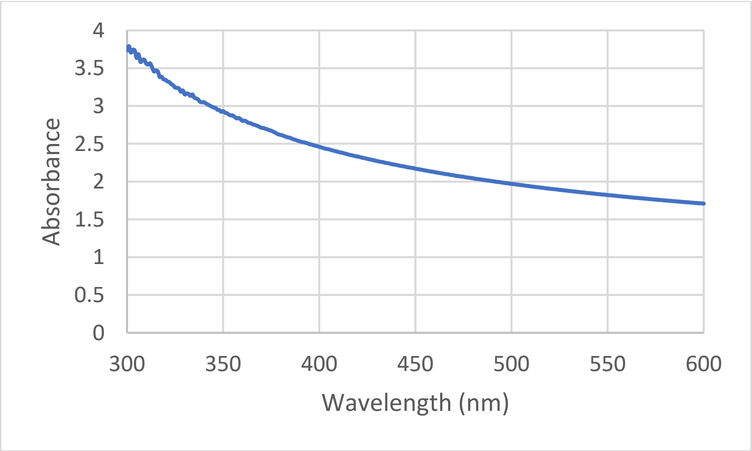

Figure S3. Example of UV-vis spectrum recorded from 300 to 600 nm of MWCNTs (feed of 0.03 mg/mL) dispersed in $PS_{26}PAA_{226}$ water solution (0.46 mg/mL, pH 5).

Polymer/MWCNT composites characterization via TGA (Section 3.5)

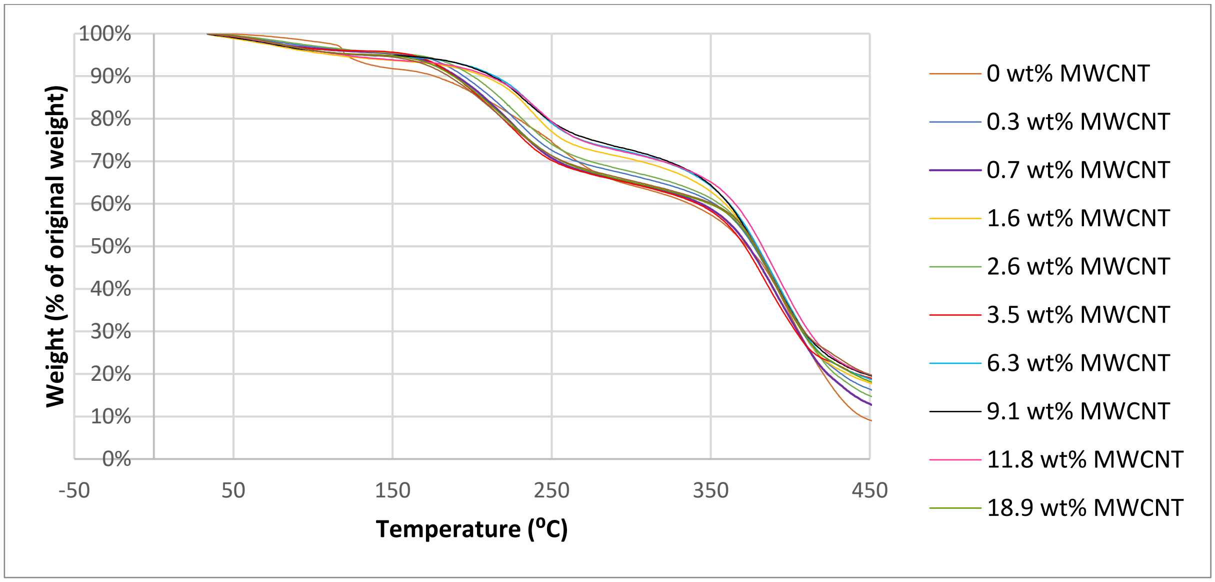

Figure S4. TGA graph: temperature starts at 25 °C and is increased with 10 °C per minute till 450 °C while nitrogen was blown over the samples. Samples weighted initially approximately 3 mg.

Calculation of surface coverage by PAA (section 3.2)

First, the calculation of the radius of gyration is explained. For this calculation only the polyacrylic acid block is considered, as the polystyrene is assumed to adsorb on the nanotube surface. The polymers are considered as individual chains forming spheres adsorbed on the nanotube surface.

Equation for Radius of gyration:

$$R_g = l_p\sqrt{\frac{N_r}{6}} \qquad (1)$$

Where:

l_p is persistence length

N_r is number of random steps (number of monomer units)

$$N_r = \frac{n_p}{l_p} \qquad (2)$$

Where:

n_p is the number of monomers in the polymer

Substituting (2) in (1):

$$R_g = \sqrt{\frac{l_p * n * l_m}{6}}$$

we can define $l_p = l_0 + l_p^{el}$

Where:

l_0 is persistence length of non-polyelectrolyte block, which is approximately 4.3 Å. [2]

l_p^{el} is the persistence length of polyelectrolyte block. This can be calculated by the following formula:

$$l_p^{el} = \frac{e^2}{16\pi\varepsilon_r\varepsilon_0 K_b T\kappa^2\alpha^2} \qquad (3)$$

where:

α is distance between charges

N_A is the Avogadro number (6.02×10^{23} mol^{-1})

I is ionic strength

e is the elementary charge

ε_0 is the permittivity of free space

ε_r is the dielectric constant of the solvent

K_b is the Boltzmann constant

T is the temperature

κ^{-1} is the Debye length

Since κ^{-1} (Debye length) is defined as:

(4)

several of these values can be crossed out by substituting (3) in (4).

$l_p^{el} = \frac{1}{32\pi\alpha^2 N_A I}$ (5)

The ionic strength and distance between the charges still have to be calculated. The ionic strength is calculated by the sum of all charges divided by two.

$$I = \frac{1}{2}\sum_{i=1}^{n} c_i z_i^2$$

Where:
C_i is the molar concentration in mol × m^{-3} of the charged specie *i*
z_i is the corresponding charge number.

The charged units in the solution are [H^+], [Na^+], [OH^-] and [AA^-] (acrylic acid). For all of these charges z=1 and the number of positive charges in the solution is equal to the negative charges. Therefore:
$I = [OH^-][AA^-]$

The [OH^-] concentration can be calculated from the pH according to:
$[OH^-] = 10^{-pOH} \times 1000$
(the factor 1000 is because of the units conversion m^3 to L).
Where:
pOH = 14–pH

The [AA^-] concentration is based on the total amount of acrylic acid units in the solution (so both charged and uncharged) and on the fraction of charged PAA units:
$[AA^-] = [AA]_{total} \times f$
Where:
f is the fraction of charged AA units

$[AA]_{total} = \frac{polymer\ feed}{M_w} \times n_p$
Where:
The polymer feed is in g*m^{-3}
During the dispersion process the concentration was 1.0 g × mL^{-1} which is 1000 g × m^{-3}
Mw is the molecular weight of the polymer in g × mol^{-1}
N_p is the number of acrylic acid monomers

The distance between the charges is.
$\alpha = \frac{l_m}{f}$
Where:
l_m is the distance between two monomers, which is 2.57 Å.[3]
f is the fraction of charged AA units

Filling in α and I in equation (5) gives:
$$l_p^{el} = \frac{1}{32\pi N_A\left(\frac{l_m}{f}\right)^2([AA]_{total}\times f + 1000\times 10^{-pOH})} = \frac{f^2}{32\pi N_A {l_m}^2([AA]_{total}\times f + 1000\times 10^{-pOH})}$$

This results in the radius of gyration as a function of *f*:
$$R_g = ((l_0 + \frac{f^2}{32\pi N_A {l_m}^2([AA]_{total}\times f + 1000\times 10^{-pOH})})\frac{n\times l_m}{6})^{\frac{1}{2}} \quad (6)$$

For the polymer PS26PAA580, which has a molecular weight of 44,675 g × mol^{-1} the relation between R_g and f is shown in Figure S5.

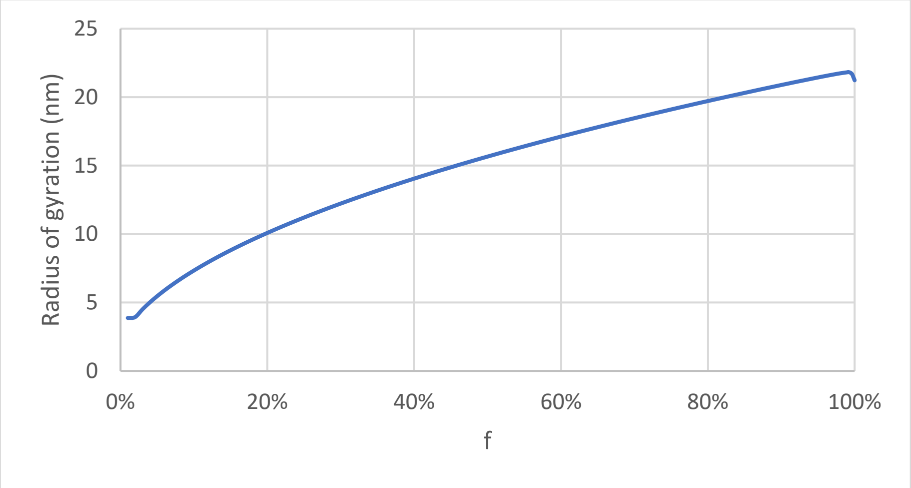

Figure S5. Graph of the relation between radius of gyration and fraction of charged AA units based on the system with PS26PAA580.

The fraction of charged acrylic acid units depends on the pH. First of all, it can be assumed that the acrylic acids in the PAA chain behave similarly as acrylic acid monomers in water. With this assumption f can be calculated based on the pK_A of acrylic acid (which is 4.25) and the following formula:

$$pH = pK_A + log_{10}(\frac{f}{1-f}) \qquad (7)$$

Filling in the f found for every pH, the relation between R_g and pH with this calculation is shown in Figure S6.

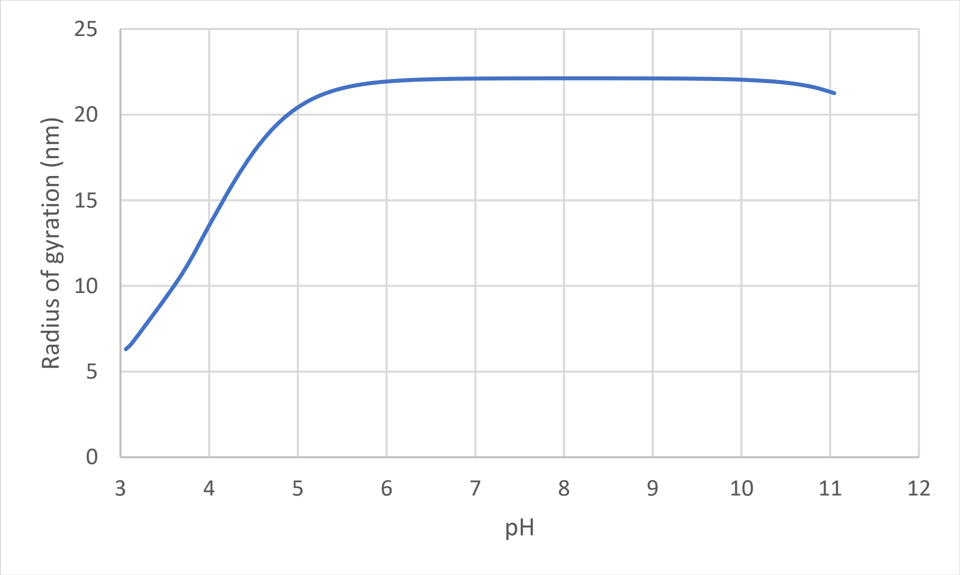

Figure S6. The relation between radius of gyration and pH with the assumption that acrylic acid behaves the same in a polymer as in individual monomers in solution. Graph is based on PS26PAA580.

However, acrylic acid behaves differently when it is in a polymer chain, because of the proximity of charges, which influences one another. Therefore, the exact relation between f and pH for a PAA chain is more complicated that equation (7) and it is a function of polymer structure and length. However, it can be approximated by the following formula: [4]

$$pH = pK_a + 4.10 f^{1/3} - \log\left(\frac{1-f}{f}\right), \quad (8)$$

This gives a different relation between R_g and pH. It is shown in Figure S7.

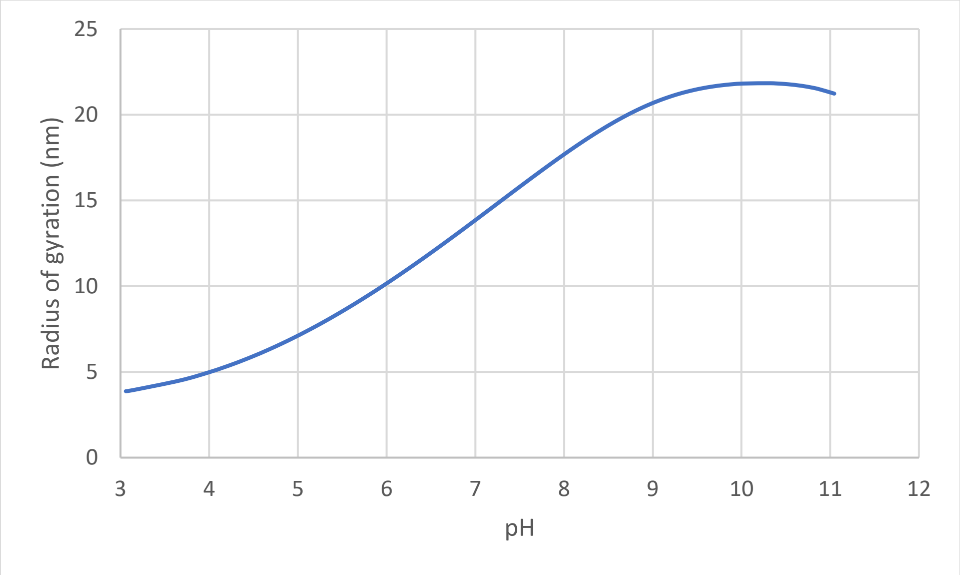

Figure S7. The relation between radius of gyration and pH for PS26PAA580, with the assumption that the proposed approximation of the relation between f and pH is correct.

The radius of gyration was calculated according to the method described above and the surface is calculated as projection of the corresponding sphere. This is multiplied by the molar concentration resulting in the total coverage.

Table S3. Calculation of the total surface coverage of the polyacrylic acid block.

Polymer	Molecular weight (g/mol)	Concentration (mg/mL)	Concentration (mmol/mL)	pH	Radius of gyration (m)	Surface area individual chain(m2)	Total surface PAA (m2)
$PS_{26}PAA_{81}$	8545	0.46	$5.38{\cdot}10^{-5}$	5	$1.26{\cdot}10^{-9}$	$2.00{\cdot}10^{-17}$	649
PAA_{454}	32715	0.46	$1.41{\cdot}10^{-5}$	5	$2.94{\cdot}10^{-9}$	$1.08{\cdot}10^{-16}$	917
$PS_{26}PAA_{226}$	18993	0.46	$2.42{\cdot}10^{-5}$	5	$2.08{\cdot}10^{-9}$	$5.45{\cdot}10^{-17}$	795
$PS_{26}PAA_{580}$	44503	0.46	$1.03{\cdot}10^{-5}$	5	$3.32{\cdot}10^{-9}$	$1.39{\cdot}10^{-16}$	864

References supporting information

1. Yadav, V.; Harkin, A. V; Robertson, M.L.; Conrad, J.C. Hysteretic memory in pH-response of water contact angle on poly(acrylic acid) brushes. *Soft Matter* **2016**, *12*, 3589–3599.

2. Cranford, S.W.; Buehler, M.J. Variation of Weak Polyelectrolyte Persistence Length through an Electrostatic Contour Length. *Macromolecules* **2012**, *45*, 8067–8082.

3. Misra, D.N. Adsorption of Polyacrylic Acids and Their Sodium Salts on Hydroxyapatite: Effect of Relative Molar Mass. *J. Colloid Interface Sci.* **1996**, *181*, 289–296.

4. Swift, T.; Swanson, L.; Geoghegan, M.; Rimmer, S. The pH-responsive behaviour of poly(acrylic acid) in aqueous solution is dependent on molar mass. *Soft Matter* **2016**, *12*, 2542–2549.
